# Padua与IMPEDE评估量表在新诊断多发性骨髓瘤患者静脉血栓栓塞症预测中的比较研究：单中心数据

**DOI:** 10.3760/cma.j.issn.0253-2727.2023.05.007

**Published:** 2023-05

**Authors:** 立娟 房, 晓东 姚, 敏秋 陆, 彬 褚, 磊 石, 珊 高, 秋晴 项, 宇彤 王, 晰 刘, 月华 丁, 远 陈, 梦真 王, 鑫 赵, 惟恺 胡, 恺 孙, 立 鲍

**Affiliations:** 北京积水潭医院，北京 100096 Department of Hematology, Beijing Jishuitan Hospital, Beijing 100096, China

**Keywords:** 静脉血栓栓塞, 多发性骨髓瘤, Padua评分, IMPEDE评分, Venous thromboembolism, Multiple myeloma, Padua score, IMPEDE score

## Abstract

**目的:**

比较两种血栓风险评估量表（Padua评分与IMPEDE评分）对中国新诊断多发性骨髓瘤（NDMM）患者6个月内发生静脉血栓栓塞症（VTE）的预测效能。

**方法:**

回顾性分析2014年4月至2022年2月北京积水潭医院NDMM住院患者的临床资料。采用Padua评分与IMPEDE评分对患者进行VTE的风险评估量化，依据受试者工作特征曲线（ROC）的曲线下面积（AUC）比较两种血栓风险评估量表对NDMM确诊后6个月内发生VTE的预测价值，并计算其灵敏度、特异度、准确率及约登指数。

**结果:**

本研究共纳入NDMM患者421例，VTE发生率为14.73％。Padua评分预测VTE的AUC为0.591（95％*CI* 0.515～0.666）。IMPEDE评分预测VTE的AUC为0.722（95％*CI* 0.648～0.796）。Padua评分预测VTE的灵敏度为100％，特异度为0％，准确率为14.7％，约登指数为0％。IMPEDE评分预测VTE的灵敏度为79.0％，特异度为44.0％，准确率为49.2％，约登指数为23.0％。

**结论:**

IMPEDE评分适用于NDMM患者6个月内发生VTE的预测。

## Abstract (en)

**Objective:**

To compare the predictive efficacy of the two thrombosis risk assessment scores（Padua and IMPEDE scores）in venous thromboembolism（VTE）within 6 months in patients with newly diagnosed multiple myeloma（NDMM）in China.

**Methods:**

This study reviewed the clinical data of 421 patients with NDMM hospitalized in Beijing Jishuitan Hospital from April 2014 to February 2022. The sensitivity, specificity, accuracy, and Youden index of the two scores were calculated to quantify the thrombus risk assessment of VTE by the Padua and IMPEDE scores. The receiver operating characteristics curves of the two evaluation scores were drawn.

**Results:**

The incidence of VTE was 14.73％. The sensitivity, specificity, accuracy, and Youden index of the Padua score were 100％, 0％, 14.7％, and 0％ and that of the IMPEDE score was 79％, 44％, 49.2％, and 23％, respectively. The areas under the curve of Padua and IMPEDE risk assessment scores were 0.591 and 0.722, respectively.

**Conclusion:**

IMPEDE score is suitable for predicting VTE within 6 months in patients with NDMM.

静脉血栓栓塞症（VTE）是包括深静脉血栓形成（DVT）与肺血栓栓塞症（PTE）在内的一组血栓栓塞性疾病，被称为“隐形的杀手”，早期识别至关重要。采用血栓风险评估量表及时准确地进行VTE风险评估，从而针对不同危险分层采取相应预防措施是防治VTE的关键。Padua评分被广泛应用于内科住院患者的VTE风险评定，是有效减少医院内VTE发生的首要环节。与普通人群相比，多发性骨髓瘤（MM）发生VTE的风险增加了9倍[1]。在MM患者中，发生VTE的患者的死亡率是未发生VTE患者的3倍[2]。为了降低中国MM患者发生VTE的风险，指导MM相关VTE的精准防治，我国血液病专家制定的《多发性骨髓瘤相关静脉血栓栓塞症防治中国专家共识（2022年版）》已正式发布，对中国MM患者的规范化管理具有重要意义[3]。在NCCN指南的2022年更新版中，已将IMPEDE评分应用于新诊断MM（NDMM）住院患者的VTE风险评定[4]–[5]，而其对中国MM患者VTE的预测效能目前尚无文献报道。因此，本研究首次探索IMPEDE评分对中国NDMM患者6个月内发生VTE的预测效能，并将其与NDMM患者Padua评分的血栓风险预测效能进行比较。

## 病例与方法

1. 病例：回顾性分析2014年4月至2022年2月北京积水潭医院421例NDMM患者的临床资料。入选标准：①经血、尿实验室检查，骨髓穿刺，病理及影像学检查确诊的NDMM患者。②具备CT肺动脉造影（CTPA）、静脉超声、动脉血气分析、血浆D-二聚体、超声心动图等完整临床资料。排除标准：妊娠；围手术期；非血栓性肺栓塞。入选患者均签署了知情同意书。

2. VTE风险分层：采用回顾性病例研究方法，搜集所有NDMM患者住院资料信息，采用Padua评分、IMPEDE评分进行VTE的诊断评估及危险分层，标准见表1、2[5]–[8]。依据指南推荐，Padua评分0～3分为低风险，≥4分为高风险；IMPEDE评分0～3分为低风险，4～6分为中风险，7分为高风险[5],[7]。

**表1:** Padua风险评估量表[6]

危险因素	分数
活动性恶性肿瘤，患者既往有局部或远端转移和（或）6个月内接受过放化疗	3
既往静脉血栓栓塞症	3
制动（患者身体原因或遵医嘱需卧床休息至少3 d）	3
有血栓形成倾向（抗凝血酶缺陷症、蛋白C或S缺乏、凝血因子Ⅴ Leiden突变、凝血酶原G20210A突变、抗磷脂综合征）	3
近期（≤1个月）创伤或外科手术	2
年龄≥70岁	1
心脏和（或）呼吸衰竭	1
急性心肌梗死和（或）缺血性脑卒中	1
急性感染和（或）风湿性疾病	1
肥胖（体质指数≥30 kg/m^2^）	1
正在进行糖皮质激素治疗	1

**表2:** IMPEDE风险评估量表[7]

危险因素	分数
正在使用免疫调节剂类药物	4
体质指数≥25 kg/m^2^	1
骨盆、髋部或股骨骨折	4
使用促红细胞生成剂治疗	1
使用多柔比星治疗	3
地塞米松治疗剂量	
大剂量地塞米松	4
小剂量地塞米松	2
种族/人种=亚洲/太平洋岛民	−3
在多发性骨髓瘤发生前有静脉血栓栓塞症史	5
有隧道式导管/中央静脉置管	2
正在进行血栓预防措施：治疗应用低分子量肝素或华法林	−4
正在进行血栓预防措施：如治疗应用低分子量肝素或阿司匹林	−3

3. VTE的诊断方法：常规于MM诊断后6个月内每个疗程化疗前进行血管彩超监测，对有单侧肢体肿胀的患者完善静脉超声以明确是否存在DVT；对有胸闷、胸痛、喘憋、低氧血症等疑似肺栓塞症状的患者完善超声心动图、CT肺动脉造影（CTPA）以明确是否存在PTE。PTE的诊断标准[9]：①CTPA检查结果符合PTE判定标准；CT仪器为Brilliance64层螺旋CT（荷兰Phillips公司）。PTE的判定标准为肺动脉内的低密度充盈缺损，部分或完全包围在不透光的血流之间，或者呈完全充盈缺损，远端血管不显影。②抗凝治疗有效。DVT诊断标准[10]–[11]：深静脉超声示深静脉内低回声部分或完全充填管腔，探头加压管腔不能变扁。

4. 随访：通过电子病历系统或电话进行随访，末次随访时间为2022年8月31日。

5. 统计学处理：采用SPSS 26.0软件进行统计学分析。正态分布计量资料以*x±s*表示，非正态分布计量资料以*M*（*Q*_1_，*Q*_3_）表示，计数资料采用例数（百分比）表示。以两种评分系统预测VTE的风险分值为检验变量，以是否发生VTE为状态变量，绘制受试者工作特征曲线（receiver operating characteristic curve, ROC），并计算曲线下面积（area under curve，AUC），对两种评分模型的AUC进行比较，并计算其灵敏度、特异度、准确率及约登指数。*P*<0.05为差异有统计学意义。

## 结果

1. 临床特征：本研究共纳入NDMM患者421例，男220例，女201例，中位年龄62.0岁。VTE组为确诊MM后6个月内发生VTE者，共62例（男33例，女29例），发生率为14.73％（62/421）。其中59例为DVT，3例为PTE。无VTE组为确诊MM后6个月内未发生VTE者，共359例（男187例，女172例）。VTE组与非VTE组患者的基线临床资料见表3。两组患者的年龄（*P*＝0.024）、既往VTE病史（*P*＝0.001）、中心静脉置管比例（*P*＝0.001）、截瘫（*P*＝0.014）、原始幼稚浆细胞比例（*P*＝0.030）及髓外病变发生率（*P*＝0.001）的差异有统计学意义，其余临床特征的差异均无统计学意义（*P*值均>0.05）。所有合并髓外病变患者均为骨旁病变，而非血行播散的髓外病变。

**表3:** VTE组与无VTE组新诊断多发性骨髓瘤患者的基线临床特征比较

特征	所有患者（421例）	VTE组（62例）	无VTE组（359例）	统计量	*P*值
中位年龄[岁，*M*（*Q*_1_，*Q*_3_）]	62.0（54.0，68.0）	63.5（57.8，69.3）	61.0（53.0，67.0）	−2.264（*Z*值）	0.024
男[例（%）]	220（52.3）	33（53.2）	187（52.1）	0.027（*χ*^2^值）	0.869
BMI（kg/m^2^，*x±s*）	24.41±3.40	24.38±3.27	24.41±3.43	0.079（*t*值）	0.948
既往VTE病史[例（%）]	34（8.1）	20（32.3）	14（3.9）	57.272（*χ*^2^值）	0.001
骨盆、股骨骨折病史[例（%）]	60（14.3）	13（21.0）	47（13.1）	2.683（*χ*^2^值）	0.101
截瘫[例（%）]	21（5.0）	7（11.3）	14（3.9）	6.093（*χ*^2^值）	0.014
ECOG评分[例（%）]				0.008（*χ*^2^值）	0.939
<2	127（30.2）	19（30.6）	108（30.1）		
≥2	294（69.8）	43（69.4）	251（69.9）		
疾病类型[例（%）]				0.297（*χ*^2^值）	0.961
IgG	172（40.9）	26（41.9）	146（40.7）		
IgA	109（25.9）	16（25.8）	93（25.9）		
轻链型	111（26.4）	15（24.2）	96（26.7）		
其他类型	29（6.9）	5（8.1）	24（6.7）		
髓外病变[例（%）]	87（20.7）	23（37.1）	64（17.8）	11.974（*χ*^2^值）	0.001
高危染色体核型[例（%）]	195（46.3）	34（54.8）	161（44.8）	2.123（*χ*^2^值）	0.145
ISS分期				1.101（*χ*^2^值）	0.577
Ⅰ期	140（33.3）	24（38.7）	116（32.2）		
Ⅱ期	118（28.0）	17（27.4）	101（28.1）		
Ⅲ期	163（38.7）	21（33.9）	142（39.6）		
DS分期[例（%）]				7.051（*χ*^2^值）	0.133
Ⅰ期	11（2.6）	1（1.6）	10（2.8）		
Ⅱ期	24（5.7）	1（1.6）	23（6.4）		
Ⅲ期	386（91.7）	60（96.8）	326（90.8）		
合并基础疾病[例（%）]	99（23.5）	14（22.6）	85（23.7）	0.035（*χ*^2^值）	0.851
应用阿司匹林预防血栓[例（%）]	118（28.0）	22（35.5）	96（26.7）	2.003（*χ*^2^值）	0.157
应用低分子量肝素预防血栓[例（%）]	55（13.1）	3（4.8）	52（14.5）	3.524（*χ*^2^值）	0.060
使用EPO[例（%）]	56（13.3）	5（8.1）	51（14.2）	1.729（*χ*^2^值）	0.189
使用免疫调节剂[例（%）]	113（26.8）	20（32.3）	93（25.9）	1.087（*χ*^2^值）	0.297
使用蒽环类药物[例（%）]	301（71.5）	46（74.2）	255（71.0）	0.260（*χ*^2^值）	0.610
中心静脉置管[例（%）]	108（25.7）	33（53.2）	75（20.9）	28.982（*χ*^2^值）	0.001
WBC[×10^9^/L，*M*（*Q*_1_，*Q*_3_）]	5.29（4.12，7.00）	5.78（4.64，7.09）	5.25（4.32，7.01）	−1.526（*Z*值）	0.127
HGB（g/L，*x±s*）	106±27	110±27	105±27	−1.334（*t*值）	0.183
PLT[×10^9^/L，*M*（*Q*_1_，*Q*_3_）]	201（105，255）	215（171，273）	198（149，252）	−1.810（*Z*值）	0.070
β_2_-微球蛋白[mg/L，*M*（*Q*_1_，*Q*_3_）]	4.20（2.77，7.28）	3.97（2.82，6.27）	4.22（2.76，7.61）	−0.738（*Z*值）	0.461
白蛋白[g/L，*M*（*Q*_1_，*Q*_3_）]	38.4（33.6，42.9）	39.4（33.8，43.2）	38.2（33.6，42.9）	−0.864（*Z*值）	0.387
肌酐[µmol/L，*M*（*Q*_1_，*Q*_3_）]	74.0（58.0，104.0）	68.0（59.0，84.5）	76.0（58.0，106.0）	−1.716（*Z*值）	0.086
血钙[mmol/L，*M*（*Q*_1_，*Q*_3_）]	2.35（2.18，2.50）	2.33（2.18，2.44）	2.36（2.18，2.50）	−0.637（*Z*值）	0.524
乳酸脱氢酶[U/L，*M*（*Q*_1_，*Q*_3_）]	173（138，213）	175（146，213）	173（137，213）	−0.529（*Z*值）	0.597
原始幼稚浆细胞比例[%，*M*（*Q*_1_，*Q*_3_）]	27.0（10.8，52.0）	23.5（5.4，44.3）	29.0（12.0，54.0）	−2.164（*Z*值）	0.030

**注** VTE：静脉血栓栓塞症；BMI：体质指数；ECOG：美国东部肿瘤协作组；EPO：促红细胞生成素

2. 治疗方案：应用VAD方案（长春新碱+阿霉素+地塞米松）18例，TCD方案（沙利度胺+环磷酰胺+地塞米松）6例，TAD方案（沙利度胺+阿霉素+地塞米松）30例，MTD方案（美法仑+沙利度胺+地塞米松）3例，BD方案（硼替佐米+地塞米松）21例，BDPACE方案（硼替佐米+地塞米松+顺铂+环磷酰胺+依托泊苷+脂质体阿霉素）13例，PAD方案（硼替佐米+脂质体阿霉素+地塞米松）179例，PCD方案（硼替佐米+环磷酰胺+地塞米松）20例，IAD方案（伊沙佐米+脂质体阿霉素+地塞米松）56例，IRD方案（伊沙佐米+来那度胺+地塞米松）33例，VRD（硼替佐米+来那度胺+地塞米松）35例，VRAD方案（硼替佐米+来那度胺+地塞米松+脂质体阿霉素）5例，DVD方案（达雷妥尤单抗+硼替佐米+地塞米松）1例，DVRD方案（达雷妥尤单抗+硼替佐米+地塞米松+来那度胺）1例。每个疗程地塞米松的使用剂量为120～160 mg。

3. 合并基础疾病情况：本研究共有99例患者合并基础疾病，其中70例患者合并糖尿病，14例患者合并慢性阻塞性肺疾病，14例患者既往有脑梗死、脑出血等脑血管病，25例合并冠心病，1例合并风湿免疫病，2例合并慢性肾脏病，2例合并肺部感染。

4. 两种评分系统对VTE的预测价值：两种评分系统预测VTE的ROC曲线见图1。Padua评分预测VTE的AUC为0.591（95％*CI* 0.515～0.666）。IMPEDE评分预测VTE的AUC为0.722（95％*CI* 0.648～0.796）。Padua评分的灵敏度为100％，特异度为0％，准确率为14.7％，约登指数为0％。IMPEDE评分的灵敏度为79.0％，特异度为44.0％，准确率为49.2％，约登指数23.0％。

**图1:**
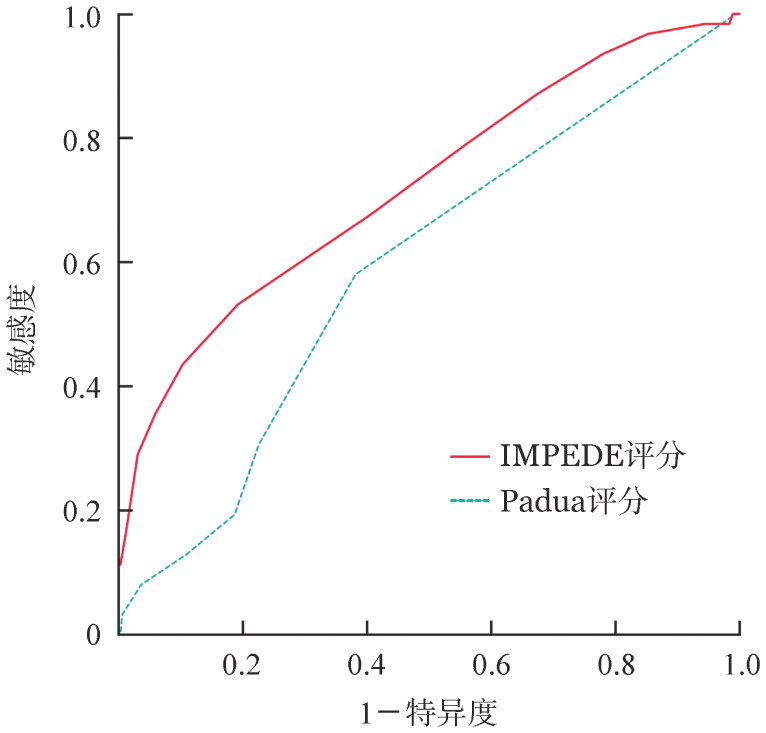
IMPEDE评分与Padua评分预测新诊断多发性骨髓瘤患者静脉血栓栓塞症的受试者工作特征曲线

## 讨论

VTE是恶性肿瘤发展及治疗过程中最常见并发症之一，已成为肿瘤患者死亡的第二大原因[12]。MM是VTE发生率最高的血液系统恶性肿瘤。MM相关VTE的发生与患者、疾病、治疗方案的危险因素相关[3],[13]。本研究通过对比患者的基线资料发现，VTE组患者的年龄、既往VTE病史比例、中心静脉置管比例、截瘫比例、原始幼稚浆细胞比例及髓外病变发生率均高于非VTE组（*P*值均<0.05）。在欧美国家NDMM患者中，症状性VTE的发病率约为10％[14]。随着免疫调节剂在MM中的广泛应用，VTE发生率也显著提高，为10％～34％[13],[15]–[16]。MM相关VTE的发生率具有种族差异，一般认为白种人的发病率高于亚洲人[17]，非裔美国人的发病率高于中国人[18]。中国MM相关VTE的数据有限，既往研究报道，NDMM患者VTE的发病率为1.2％～5.5％[18]–[24]。在MM新药时代，中国NDMM患者VTE的发生率尚缺乏大样本统计，本研究结果显示，421例接受以化疗为基础治疗的NDMM患者6个月内VTE的发生率为14.73％（62/421），显著高于国内早期文献报道的VTE发生率。我们分析可能的原因包括：①本研究DS分期Ⅲ期患者多，肿瘤负荷高；②MM骨病导致的瘫痪卧床患者比例较高；③重视VTE影像学筛查，血管超声检出更多隐匿性无症状VTE患者。

Padua评分模型由意大利Padua大学血栓栓塞中心Barbar等[6]研究开发，目前国内也普遍应用Padua模型进行内科住院患者VTE的风险评估。本研究显示，针对NDMM患者VTE的风险评估，Padua评分并不适用，因为MM一经诊断，治疗方案基本均包含激素类药物，导致患者Padua评分均≥4分，为高危患者，提示该评分预测模型对NDMM患者发生VTE的预测价值有限，值得血液内科医师关注。近年来，Sanfilippo等[7]针对VACCR数据库（Veterans Administration Central Cancer Registry）中4 446例NDMM患者开发了IMPEDE VTE评分模型并进行内部验证，同时应用SEER数据库中4 256例NDMM患者进行外部验证，已成为现阶段最优的NDMM患者VTE预测模型[4],[25]。2022年NCCN已将IMPEDE模型纳入指南[5]，用于VTE的风险评估。本研究首次在中国NDMM患者中证明IMPEDE预测模型可较好地预测NDMM患者6个月内发生VTE的风险，可用于指导MM相关VTE的精准分层预防。

本研究证明IMPEDE评分适用于中国NDMM患者6个月内发生VTE的风险评估，其预测效能优于内科血栓风险评估量表Padua评分。本报告为单中心回顾性研究，可能存在样本偏倚。此外，本研究始于《多发性骨髓瘤相关静脉血栓栓塞症防治中国专家共识（2022年版）》[3]发布之前，与共识中拟定的中国MM患者VTE风险分层的积分系统相比较，IMPEDE评分内容不够详细，在前期工作基础上如何优化VTE风险评估模型值得进一步探讨。
